# Imaging gold nanoparticles in mouse liver by laser ablation inductively coupled plasma mass spectrometry

**DOI:** 10.1038/s41598-017-03275-x

**Published:** 2017-06-07

**Authors:** Qing Li, Zheng Wang, Jiamei Mo, Guoxia Zhang, Yirui Chen, Chuchu Huang

**Affiliations:** 1 Shanghai Institute of Ceramics, Chinese Academy of Sciences, Shanghai, 200050 China; 2 University of Chinese Academy of Sciences, Beijing, 100049 China

## Abstract

Imaging the size distribution of metal nanoparticles (NPs) in a tissue has important implications in terms of evaluating NP toxicity. Microscopy techniques used to image tissue NPs are limited by complicated sample preparation or poor resolution. In this study, we developed a laser ablation (LA) system coupled to single-particle inductively coupled plasma mass spectrometry (SP-ICP-MS) for quantitative imaging of gold (G)NPs in tissue samples. In this system, GNPs were ablated but did not disintegrate and integrate under optimised operation conditions, which were verified by characterising LA particles by scanning electron microscopy. The feasibility of imaging size distributions in tissue was validated using reference GNPs 60 and 80 nm in size on matrix-matched kidney. A transport efficiency of 6.07% was obtained by LA-SP-ICP-MS under optimal conditions. We used this system to image 80-nm GNPs in mouse liver and the size distribution thus obtained was in accordance with that determined by nebuliser SP-ICP-MS. The images revealed that 80-nm GNPs mainly accumulate in the liver and did not obviously aggregate. Our results demonstrate that LA-SP-ICP-MS is an effective tool for evaluating the size distribution of metal NPs in tissue.

## Introduction

In the past decade, metal nanoparticles (MNPs) have been widely used in biotechnology, manufacturing, and other areas due to their unique properties. However, their intentional or inadvertent release into the surroundings raises concerns about potential risks to the environment as well as to human health^1^. MNPs reportedly have higher toxicity than metal ions of the same elements^2, 3^. However, the mechanism of toxicity of MNPs has not yet been elucidated. Many studies have shown that the toxic properties are influenced by particle composition, size, and shape^4, 5^. Various methods have been used to obtain size information such as dynamic light scattering^6^, atomic force microscopy^7^, scanning electron microscopy (SEM) and transmission electron microscopy (TEM)^8, 9^, and fluorescence imaging^10, 11^. However, these methods are time-consuming, costly, or involve complicated sample preparation procedures, including time-consuming labelling processes or requiring tissue samples to be prepared as solutions. Nonetheless, imaging the size distribution of NPs in tissues is necessary in order to better understand the hazards associated with their presence in organisms^12^. It is therefore of considerable interest to develop simple and time-consuming approaches for imaging MNP size distribution in tissues.

Single-particle inductively coupled plasma mass spectrometry (SP-ICP-MS) is considered as a promising analytical approach for the detection and characterisation of MNPs at low concentrations, as it allows simultaneous determination of particle size distribution and number^13–16^. However, it cannot be used for imaging of MNP size distribution in tissues since its mode of sample introduction restricts the imaging analysis of solid samples. Recently, 56 and 86 nm gold (G)NPs on a suitable absorbing plastic surface were directly analysed by substrate-assisted laser desorption (SALD) SP-ICP-MS^17^. Another study described a method for quantitative characterisation of GNPs involves coupling thin layer chromatography with laser ablation (LA)-ICP-MS and chemiluminescence^18, 19^. However, these approaches do not solve the problem of direct determination of size distribution in solid samples, namely biological tissues.

**Figure 1:**
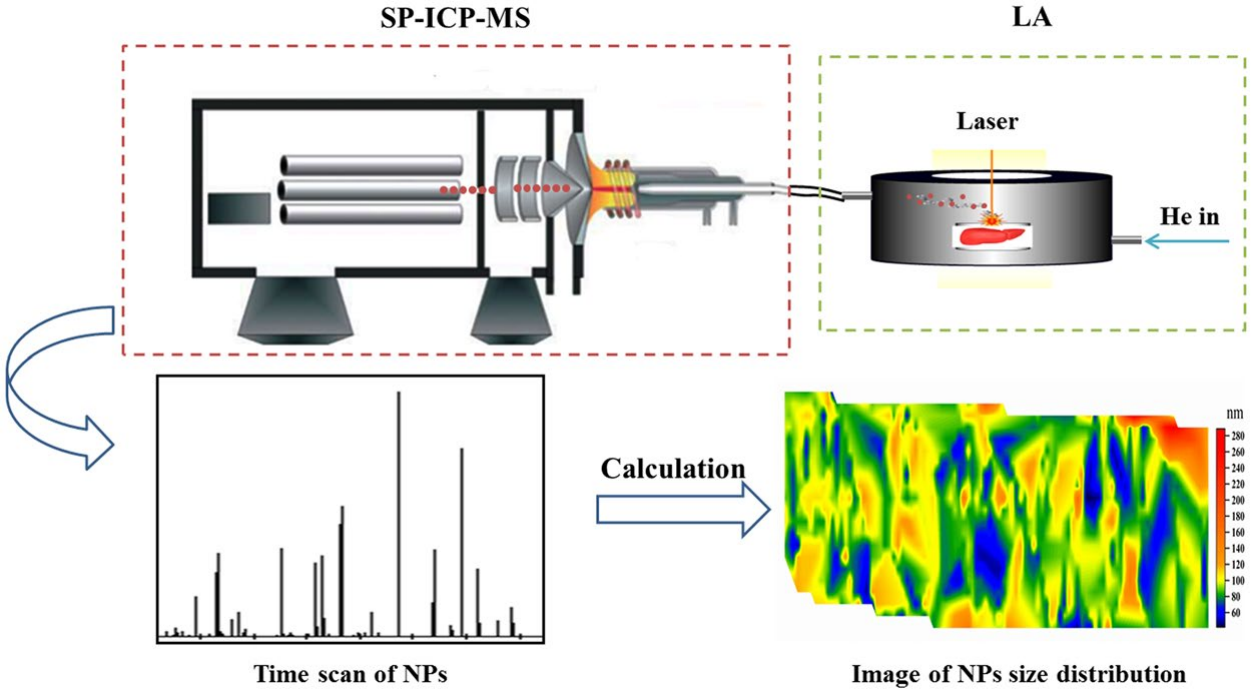
Schematic illustration of LA-SP-ICP-MS. Particles in mouse liver were ablated; LA aerosols were introduced into the ICP-MS instrument by the carrier gas (He) and ionised; and pulse signals were detected. Each pulse represents a single particle event. Images of NP size distribution were obtained by calculation.

In this study, we developed a method for imaging GNP size distribution in tissues using LA coupled with SP-ICP-MS. To the best our knowledge, LA-SP-ICP-MS has not been previously used to image GNP size distribution in tissues. A schematic illustration of LA-SP-ICP-MS is shown in Fig. 1. Laser fluence was optimised in order to reduce particle disintegration during the laser ablation process. We used this method to evaluate the transport efficiency of 60- and 80-nm GNPs on matrix-matched kidney. The distribution of intravenously injected 80-nm GNPs in the liver was also examined.

## Results and Discussion

### Theoretical analysis

SP-ICP-MS employs the well-established, widespread, and highly sensitive method for elemental analysis, ICP-MS, but is carried out by a time-resolved analysis with shorter dwell times than that of typical ICP-MS analysis^20, 21^. Single particles enter the ICP-MS plasma and, once ionised, move through the mass analyser to the detector, detect pulse signals; each pulse represents a single-particle event that can provide valuable information about particle size and size distribution^14, 15, 22^. In SP-ICP-MS, pulse frequency is directly related to NP concentration, and signal intensity is proportional to particle mass, which can be translated into size by assuming a spherical shape. The equations for determining both particle number concentration and particle size by SP-ICP-MS are shown in the Supplementary Information.

LA-SP-ICP-MS analysis of GNPs is mainly affected by laser fluence, which ensures that particles will not disintegrate and integrate into the LA process. In an ideal case, intact NPs should be ablated without disintegration and all or most NPs will be ablated as long as the laser fluence is as low as possible. A fluence of 0.1–1.0 J·cm^−2^ was previously shown to provide sufficient energy for GNP ablation, while a fluence > 1 J·cm^−2^ resulted in GNP disintegration^17^. According to the linear output of laser energy for different energy levels (Fig. S2), a 5% energy level, low laser fluence, and large laser spot would meet the requirement of a laser fluence < 1.0 J·cm^−2^. Thus, under these conditions, GNPs can be ablated without integration. This was tested by ablating 80-nm GNPs on the kidney.

**Figure 2:**
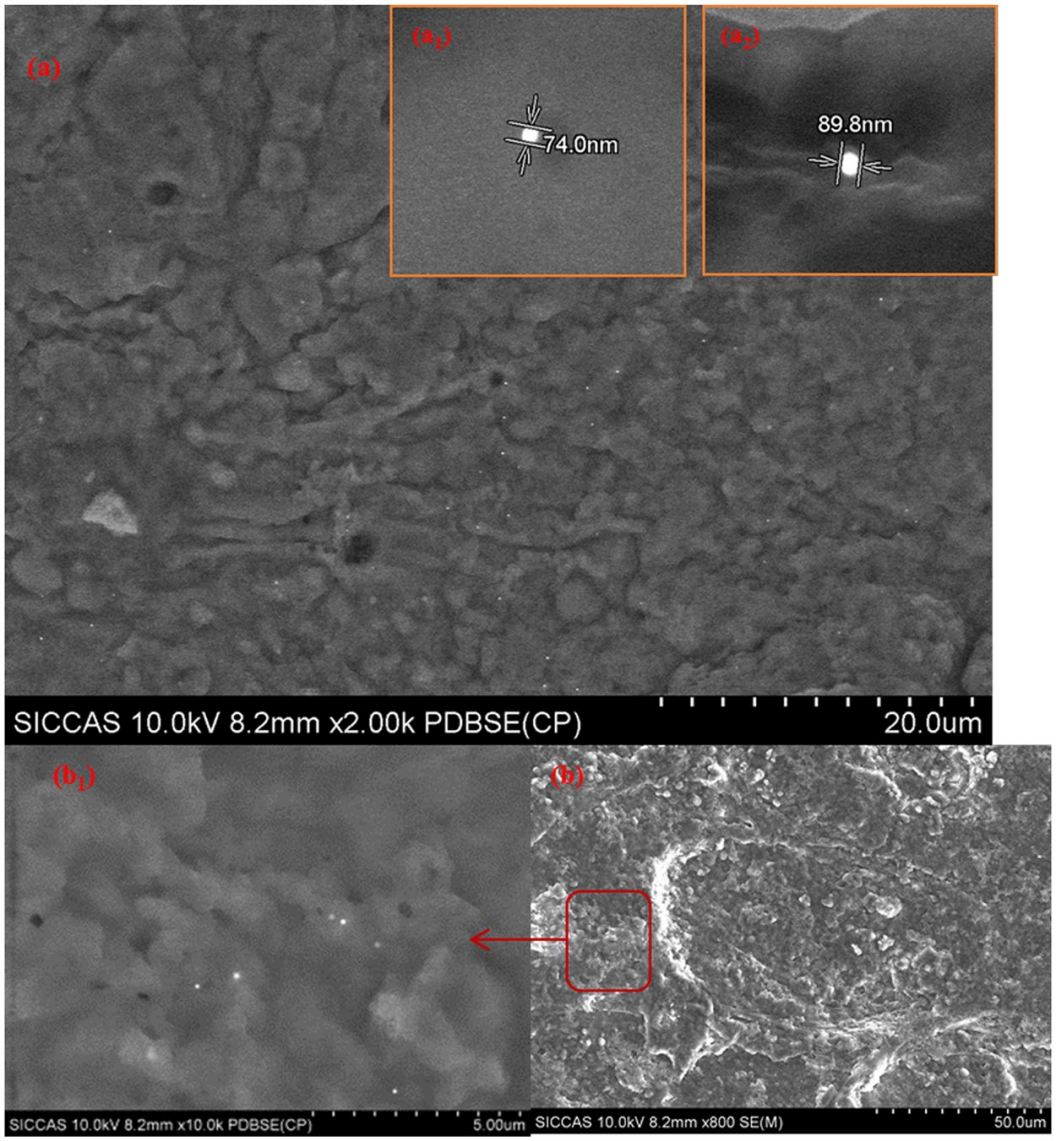
SEM images of GNPs on kidney (**a**) and determination of GNP size (a1 and a2). (**b**) Images of ablated GNPs on kidney; the area outlined in red represents the LA product (shown at higher magnification in b1).

GNPs about 80 nm in size were uniformly distributed on the matrix-matched kidney sample without obvious aggregation (Fig. 2a). Samples prepared using this approach met the requirements of LA-SP-ICP-MS analysis and prevented the simultaneous entry of too many particles into the detector during signal capture. The size of GNPs in LA residues remained about 80 nm, and particles were distributed near the ablation hole on the kidney (Fig. 2b and b1), demonstrating that LA did not cause GNP disintegration or integration. These results indicate that a laser fluence of 5% energy level and a large laser spot met the LA-SP-ICP-MS analysis requirements.

### Determination of transport efficiency

In LA-SP-ICP-MS experiments, transport efficiency is determined by transport from the ablated cell to the ICP and ionisation efficiency in plasma^23^. Factors affecting transport efficiency subjected to LA include inertial impact, laminar and/or turbulent diffusion, gravitational settling, and electrostatic attraction as well as sample preparation^17^.

Transport efficiency is calculated as the ratio of the amount of analyte entering the plasma to the amount of aspirated analyte. To assess the transport efficiency of LA-SP-ICP-MS, droplets (6.8 μL) of 1.10 × 10^8^ GNP·mL^−1^ were deposited onto a dry area of a pig kidney slice and allowed to dry prior to analysis under optimal conditions. The number of NP events was determined as 45,403 ± 3219 GNPs. A transport efficiency of 6.07% ± 0.43% was calculated as the ratio of the total number of detected GNPs to the exact number of particles (748,000) deposited onto the kidney slice.

The transport efficiency was far lower than the reported value of LA-ICP-MS, for which aerosol transport efficiency exceeds 80% for particle sizes in the range of 5 nm–3 μm^24^. However, the value was higher than the 2.4% obtained for nebuliser SP-ICP-MS under optimal conditions in our work. These may be explained as follows. Firstly, whereas the transport efficiency of LA-ICP-MS refers to aerosol transport from the ablation cell to ICP, that of LA-SP-ICP-MS includes transport loss from the tube, LA interface, and ICP-MS, all of which affect GNP transport into plasma in addition the transformation of aerosol into ions. Secondly, GNPs within the matrix-matched sample were not ablated since they had penetrated the tissue. Thirdly, some GNP signals may have been lost as a result of long dwell times, which may also have occurred for nebuliser SP-ICP-MS. On the other hand, the higher transport efficiency compared to nebuliser SP-ICP-MS is likely because direct sampling of the LA method minimised transport loss from the sample to ICP as compared to solution sampling owing to low nebulisation efficiency.

### Calibration of GNP concentration

**Figure 3:**
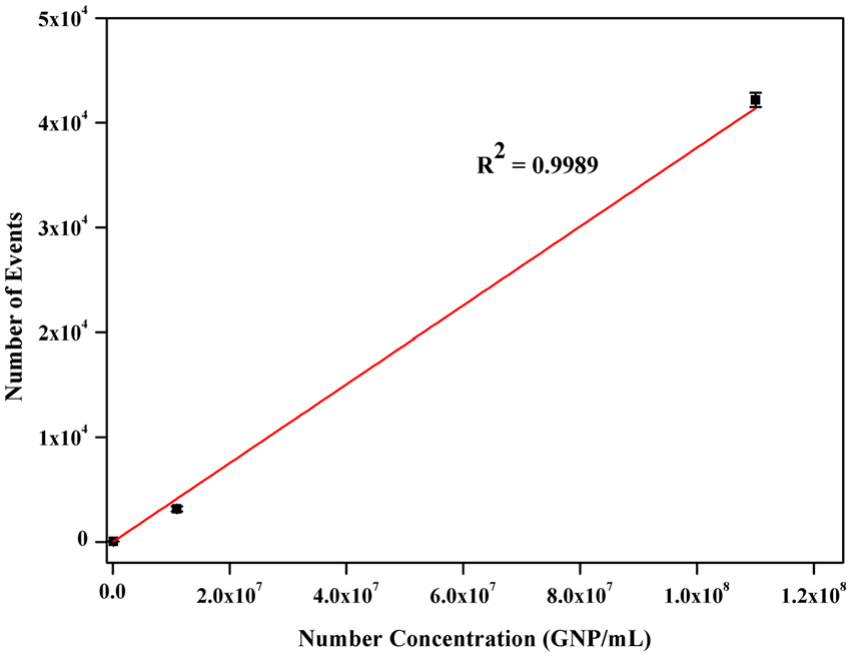
Calibration plot of number of NPs vs. GNP concentration.

The pulse frequency in SP-ICP-MS—i.e., the number of NP events—was directly related to NP concentration, which was independent of NP size. To validate our approach, we generated a calibration plot of the number of GNP events as a function of GNP concentration (Fig. 3).

Droplets (6.8 μL) with 1.10 × 10^8^, 1.10 × 10^7^, 1.10 × 10^5^ GNP·mL^−1^ (80 nm) were deposited on kidney slices and analysed. The correlation coefficient was 0.9989, indicating good linearity. The slope of 0.057, which is in good agreement with the transport efficiency of 0.061 reported above, underscored the reliability of our approach. The value of transport efficiency was lower than that of SALD-SP-ICP-MS (0.61)^17^ and higher than that obtained by SP-ICP-MS (0.013, 0.0156, and 0.0398)^15, 25, 26^. This is likely due to differences in transport efficiency of the sampling device—i.e., a direct sampling device (i.e., LA and SALD) reduces transport loss in contrast to a solution sampling device with 3%–5% nebulisation efficiency (i.e., nebuliser and spray chamber)^27^.

### Analysis of 60- and 80-nm GNPs

**Figure 4:**
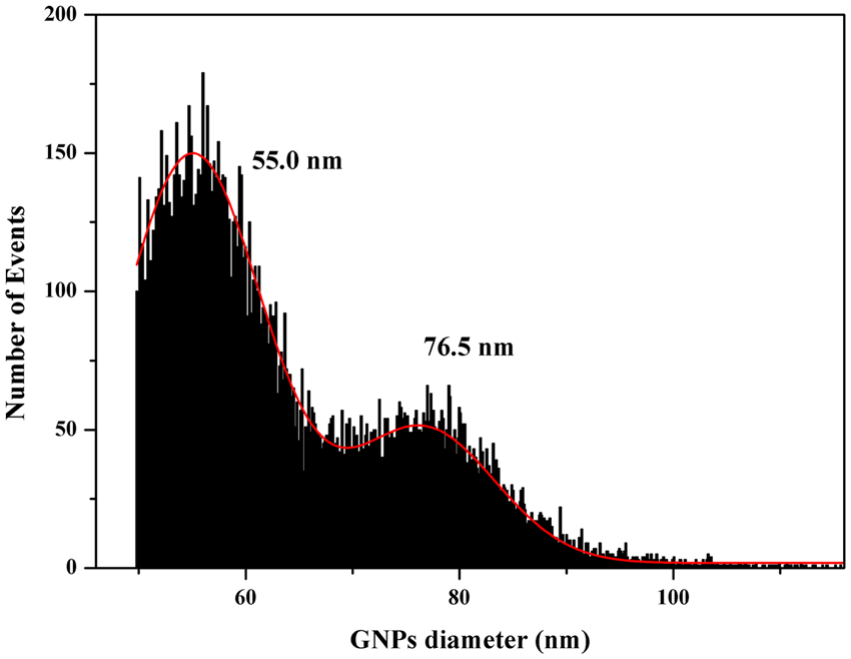
Size distribution histograms of 60- and 80-nm GNPs obtained by LA-SP-ICP-MS under optimal conditions.

To further evaluate the performance of LA-SP-ICP-MS for quantitative analysis of GNPs, a mixed sample of GNPs of the two sizes was analysed. Droplets (6.8 μL) with 1.10 × 10^8^ 60- and 80-nm GNPs were deposited on a kidney slice and measured under identical conditions. The size histogram revealed mean GNP sizes of 55.0 and 76.5 nm (Fig. 4) that were consistent with the mean sizes of 56.0 and 79.2 nm reported by the manufacturer.

### Biodistribution of GNPs in mouse

**Figure 5:**
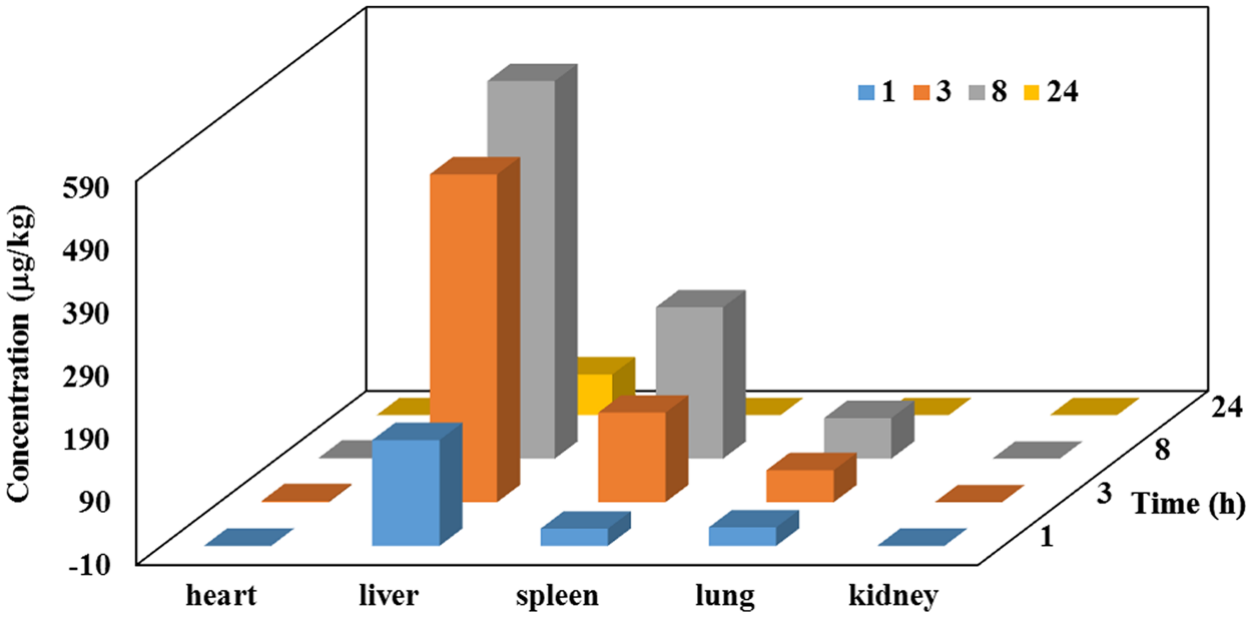
Au concentration in mouse tissues at different time points, as determined by LA-ICP-MS. Heart, liver, spleen, lung, and kidney tissue were harvested 1, 3, 8, and 24 h after intravenous injection of GNPs.

It has great significance to study GNPs in tissues whether agglomeration or degradation for toxicology of nanoparticles. We therefore assessed GNP biodistribution after intravenous injection in mice by LA-ICP-MS by multi-line scanning of the heart, lung, spleen, liver, and kidney, which are the major metabolic organs. We found that the Au content of these tissues changed over time (Fig. 5). Additional details can be found in the Supplementary Information, including the detection limit of Au.

Au content increased in the first 8 h but was diminished at 24 h in the liver, spleen, and lung. The content in kidney was markedly lower than that in the liver and spleen. Particles with a diameter < 8 nm may be filtered by the glomerular capillary membrane of the kidneys into the renal tubules, and consequently cleared via the urine^28^. Since the size of GNPs was much larger than the threshold for renal excretion, they were not cleared from the kidney.

Au concentration in the spleen was lower than that in the liver at each time point examined (Fig. 5). Endothelial cells that compose blood vessel walls are classified as continuous, fenestrated, or discontinuous. The continuous endothelium morphology is observed in arteries, vessels, and lungs. In contrast, the fenestrated endothelium occurs in glands, digestive mucosa, and kidney. Fenestrae have 60-nm pores exhibiting octagonal symmetry. A discontinuous endothelium is found in the liver and is composed of fenestrae of 50–100 nm^29–31^; this accounts for the accumulation of GNPs primarily in the liver. GNPs may accumulate in lungs due to factors such as composition, size, core properties, and surface charge; it was previously reported that polyethylene glycol-coated GNPs accumulated in the lungs^32^.

### Imaging analysis of GNPs in mouse liver

**Figure 6:**
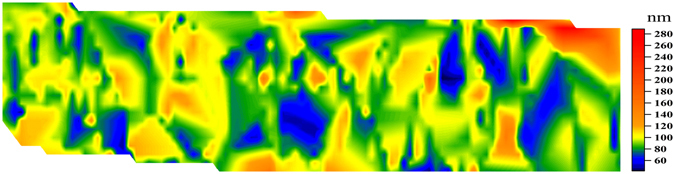
Magnified image of GNP size distribution on liver 8 h after intravenous injection.

We investigated the size distribution of GNPs in the liver by LA-SP-ICP-MS 8 h after intravenous injection. The 80-nm GNPs mostly accumulated in the liver after 8 h. Under optimal conditions, we ablated a piece of liver 8 h after GNP injection and generated a size distribution map (Fig. S4). The size distribution of GNPs was about 80 nm (Fig. 6). We validated the imaging results using the nebuliser SP-ICP-MS method, since there were too few GNPs in the liver for characterisation by SEM (Fig. S5)^15^. The mean diameter of particles in the water-leached solution from liver 8 h after intravenous injection was 75.0 nm, as determined by SP-ICP-MS (Fig. S6). These results are consistent with the imaging data obtained by LA-SP-ICP-MS, confirming the reliability of this method.

## Conclusions

In the present study, we developed a method for quantitative imaging of GNPs in tissue samples and determination of particle size distribution using LA and SP-ICP-MS. Under optimal conditions, 60- and 80-nm GNPs were detected on the matrix-matched kidney. The LA-SP-ICP-MS method was used to image 80-nm GNPs that were accumulated in the liver, and the results were in accordance with those obtained by nebuliser SP-ICP-MS. GNPs in the liver 8 h after intravenous injection did not agglomerate and maintained a size range of 80 nm. Our results highlight the potential for using LA-ICP-MS for NP toxicity investigations, thereby circumventing the limitations of SEM analysis.

## Methods

### Chemicals

A certified Au (III) reference element standard (1 g·L^−1^) in 2% nitric acid was purchased from National Analysis Center for Iron and Steel, China Iron & Steel Research Institute Group (Beijing, China). Double-distilled water (DIW; 18.25 MΩ·cm) obtained using a Milli-Q system (Millipore, Bedford, MA, USA) was used for experiments. GNPs (BBI Solutions, Cardiff, UK) had mean core diameters of 56.0 ± 0.5 and 79.2 ± 6.34 nm according to TEM characterisation by the manufacturer. GNP concentration determined based on the Au mass fraction and mean diameter provided by the manufacturer were 2.6 × 10^10^ and 1.1 × 10^10^ GNP·mL^−1^, respectively.

### Sample preparation

Experiments were carried out with 80-nm GNPs unless otherwise stated. The GNP suspension was diluted with DIW. Between each dilution step, NP suspensions were sonicated for 1 min.

### Matrix-matched sample preparation

Pig kidney purchased from a local market was used to prepare matrix-matched samples. The tissue was sliced and dried in a vacuum oven (DZF; Shanghai JingHong Laboratory Equipment Co., Shanghai, China). The slices were further cut into pieces with dimensions of about 0.5 × 0.5 cm. The certified Au (III) reference element standard (1 g·L^−1^) was diluted with water to specific concentrations and 6.8-μL droplets of GNP suspension or Au (III) standard solution were added to each kidney sample using a conventional micropipette (Transferpette S; Brand, Wertheim, Germany). Droplets were allowed to dry at room temperature prior to analysis. GNPs on kidney tissue were characterised by field emission SEM using a JSM-6700F microscope (JEOL, Tokyo, Japan).

### Nebuliser SP-ICP-MS

GNPs were analysed by nebuliser SP-ICP-MS. The GNP suspension was diluted with DIW to concentrations of 2.60 × 10^4^ and 1.10 × 10^5^ GNP·mL^−1^ for 60- and 80-nm particles, respectively. The optimised conditions are shown in Supplementary Information Table S1. The transport efficiency was calculated as 2.4% and mean particle diameters were 55.2 and 72.7 nm of 60- and 80-nm GNPs, respectively (Fig. S1).

### *In vivo* biodistribution studies

Female Balb/c mice (7 weeks old) with average weight of 20 g were purchased from the Laboratory Animal Center of Shanghai Medical College of Fudan University. All animal experiments were conducted under protocols approved by the Laboratory Animal Centre of Fudan University, China. GNPs at a total dose of 150 μL (57 mg Au/kg dose, in 150 μL saline) were administered to mice by intravenous injection. Tissues were harvested at 1, 3, 8, and 24 h post-injection. The optimised conditions for determining GNP biodistribution are shown in Table 1.

**Table 1:** Optimised conditions for LA-SP-ICP-MS and quantitative analysis.

LA parameters	LA-SP-ICP-MS values	Quantitative analysis values	ICP-MS parameters	LA-SP-ICP-MS values	Quantitative analysis values
Laser wavelength (nm)	213	213	RF power (W)	1400	1400
Laser energy (%)	5	10	Sampling depth	150	150
Laser frequency (Hz)	1	20	Signal acquisition mode	Time-resolved analysis	Time-resolved analysis
Ablation spot size (μm)	200	100	Dwell time (ms)	1	10
Carrying gas (He) (l/min)	0.7	0.7	Carrying gas (Ar) (l/min)	0.7	0.7
Scan rate (μm/s)	69	20	Isotopes	197Au	197Au

### LA-SP-ICP-MS measurements

A LSX-213 laser system (Teledyne Cetac, Omaha, NE, USA) was used to ablate GNP samples. The aerosol from LA was transported into an ICP quadrupole mass spectrometer (X Serials 2; Thermo Fisher Scientific, Waltham, MA, USA). The signal for the 197Au isotope was measured with an integration time of 1 ms, which was the lowest possible value for the instrument software. Optimised conditions for LA-SP-ICP-MS are shown in Table 1.

### Data processing

Data were processed using Microsoft Excel to calculate particle size and determine the size distribution. Signals were isolated from the background using an iterative algorithm to qualify a given intensity as a signal^33^. For LA-SP-ICP-MS data processing, reference GNPs were used to calibrate signal intensity. The intensity histogram of reference GNPs was fitted with a normal distribution curve, which was used to determine the maximum signal intensity corresponding to the mean diameter of reference GNPs. A more detailed description of this process can be found in the Supplementary Information.

## Electronic supplementary material

Supplementary Information
