# Supplementary material for: Imaging gold nanoparticles in mouse liver by laser ablation inductively coupled plasma mass spectrometry
Source: Sci Rep. 2017 Jun 7;7:2965. doi: 10.1038/s41598-017-03275-x (PMC5462741; doi:10.1038/s41598-017-03275-x)
Supplement: Supplementary file 1 — Supplementary Information [file 41598_2017_3275_MOESM1_ESM.pdf]

Supplementary Information

Imaging gold nanoparticles in mouse liver by laser ablation inductively coupled plasma mass spectrometry

Qing Li[1,2], Zheng Wang[1,*], Jiamei Mo[1], Guoxia Zhang[1], Yirui Chen[1], Chuchu Huang[1]

[1] Shanghai Institute of Ceramics, Chinese Academy of Sciences, Shanghai, 200050, China.

[2] University of Chinese Academy of Sciences, Beijing, 100049, China.

*Correspondence and requests for materials should be addressed to Z.W. (email:wangzheng@mail.sic.ac.cn)

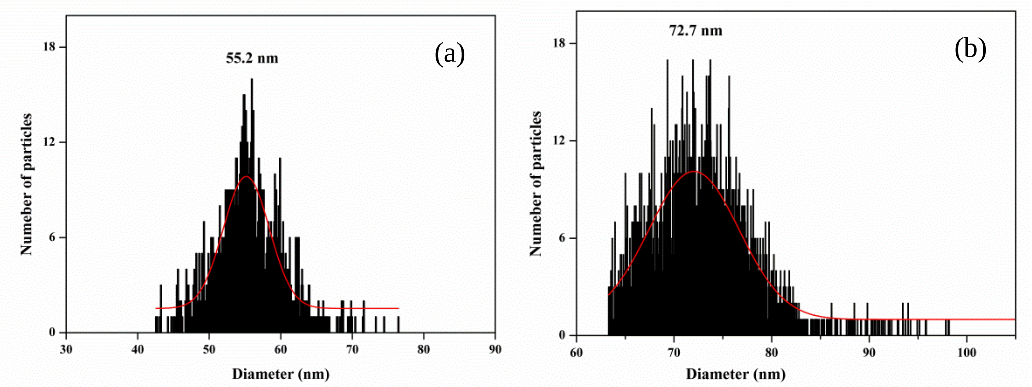

Figure S1. Size distribution histograms of 60-nm (a) and 80-nm (b) GNPs obtained by nebuliser SP-ICP-MS. The red solid line represents the Gaussian size distribution fitting.

Table S1. Optimal conditions for nebuliser SP-ICP-MS

**Instrument settings**			
Sample flow	0.36 ml/min	Signal acquisition mode	Time-resolved analysis
Dwell time	5 ms (60 nm); 3 ms (80 nm)	Isotopes	197Au
RF power	1400 W	Carrying gas (Ar)	0.88 l/min
Sampling depth	150		
**Determination of nebulisation efficiency**			
Element density	19.32 g/ml	Concentration (mass)	56.6 mg/l
Standard particle diameters	56.0 ± 0.5 nm; 79.2 ± 6.34 nm	Concentration (particle number, particles/ml)	2.6×10^4 (60 nm); 1.1×10^5 (80 nm)
Calculated transport efficiency	2.4%		
Calculated mean size	55.2 nm; 72.7 nm		

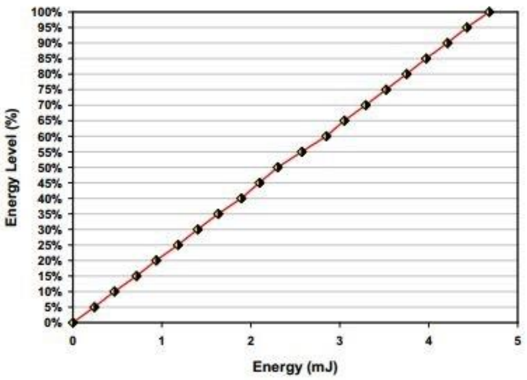

Figure S2. LSX-213 linear output of laser energy for different energy levels (data from manufacturer).

Determination of Au concentration in tissues by LA-ICP-MS

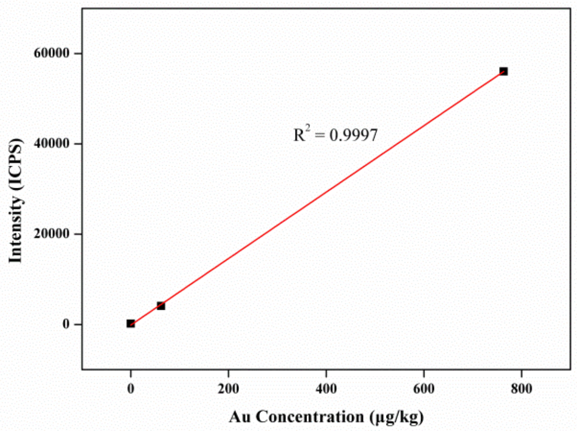

Figure S3. Calibration curve for Au obtained with matrix-matched standards.

Matrix-matched standards were prepared from a slice of kidney tissue spotted with droplets (6.8 μL) of known Au standard solution (~100 and ~1000 μg/L). The actual concentrations of matrix-matched standards were determined by a typical digestion ICP-MS method. The detect limit was determined according to the IUPAC 3s criterion (Vanhaecke et al., 2010). The limit of detection of Au was 0.02 μg/kg, which was three orders of magnitude higher than that obtained by other LA-ICP-MS methods (Vanhaecke et al., 2010).

Table S2. Au content of heart, liver, spleen, lung, and kidney after intravenous injection of GNPs at different time points

Tissue (μg/kg)	Time point (h)			
	1	3	8	24
Heart	< 0.2	1.9	1.7	< 0.2
Liver	168.4	520.9	1913.5	64.6
Spleen	27.3	142.6	240.0	< 0.2
Lung	29.4	51.0	64.6	< 0.2
Kidney	< 0.2	< 0.2	< 0.2	< 0.2

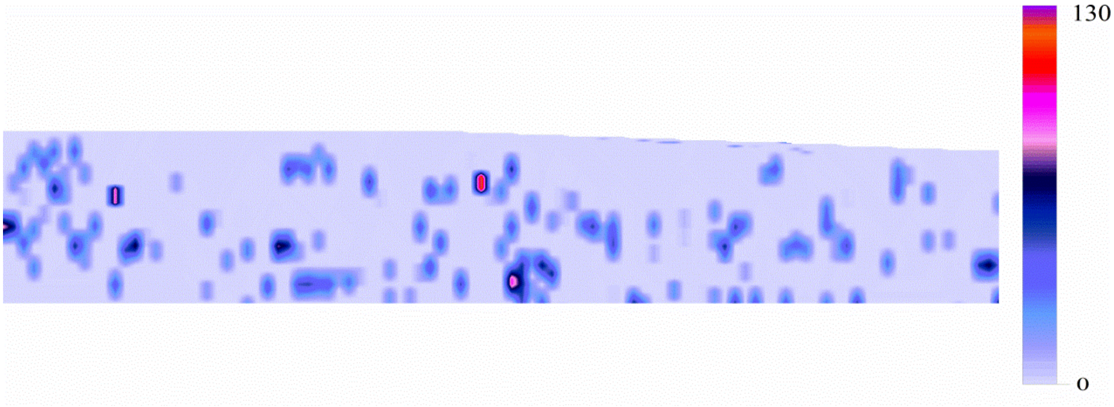

Figure S4. Imaging GNP size distribution in liver 8 h after intravenous injection.

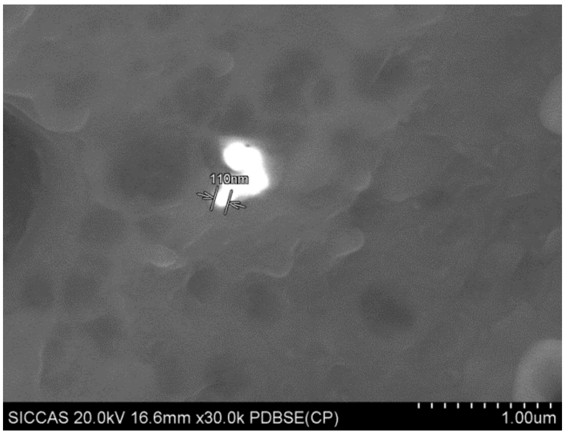

Figure S5. SEM image of GNPs on liver 8 h after intravenous injection. GNPs on the liver were too few to be characterised by SEM.

Digestion of the imaged liver sample

The liver was cut into small pieces with a surgical knife 8 h after intravenous injection of GNPs for the validation study. The pieces were transferred to a 10-mL polyethylene tube. A 5-ml volume of water was added to the tube, which was placed in an ice bath and sonicated for 5 min. The digested solution was analysed by SP-ICP-MS.

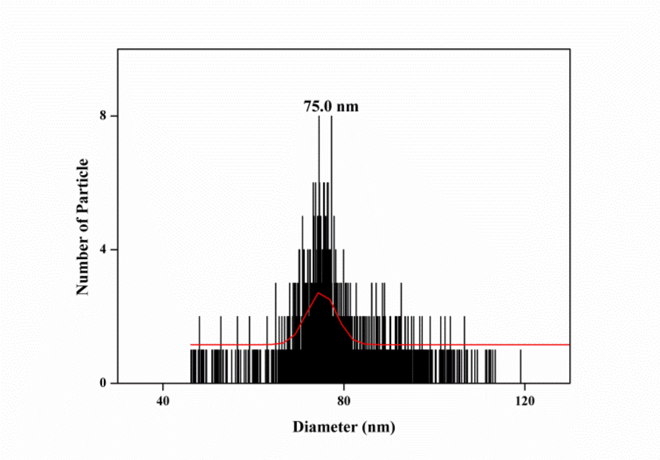

Figure S6. Size distribution of GNPs on liver 8 h after intravenous injection, as determined by SP-ICP-MS.

Data processing

For SP-ICP-MS, an iterative algorithm was applied to distinguish between dissolved background and pulses. The 3-s value was first calculated and added to the average; higher values were attributed to NPs and were therefore retained. This process was repeated with the remaining data until no more pulses could be differentiated. The particle size and number concentration was calculated as follows:

$$C_p = \frac{N_p}{\eta_n} \times \frac{1000}{V}$$

where C_p = particle number concentration (L^{-1}), N_p = number of particles detected in the time scan (min^{-1}), η_n = nebulisation efficiency, and V = sample input flow (mL/min). The same formula was used to calculate nebulisation efficiency after measuring the reference NPs, in which case the particle number concentration (C_p) was known and the nebulisation efficiency was calculated from the observed number of particles (N_p) in the time scan, as follows:

$$m_p = \frac{I_p t_d}{RF_{ion} \times f_m} \times \frac{V\eta_n}{60}$$

where m_p = particle mass (ng), I_p = particle signal intensity in the sample (cps), RF_{ion} = ICP-MS response factor from the calibration curve of ionic standards (cps/μg/L), f_m is the mass fraction of analytical element (f_m = 1 when the analytical element is Ag or Au), and t_d = dwell time (s); in addition,

$$d_p = \sqrt[3]{\frac{6m_p}{\pi\rho_p}} \times 10^4$$

where d_p = particle diameter in the sample (nm) and ρ_p = particle density (g/mL).

For LA-SP-ICP-MS data processing, the size distribution was calculated based on the N_p value. The intensity histogram of reference GNPs was fitted with a normal distribution curve that was used to determine maximum signal intensity ($S_{R,max}$) corresponding to the mean diameter of the reference GNP (d_R). To obtain the sample size distribution, signal intensity (S) was transformed to diameter (d) using the following formula.

$$d = d_R \times \sqrt[3]{\frac{S}{S_{R,max}}}$$

Reference:

Vanhaecke, F., Resano, M., Koch, J., Mcintosh, K. & Günther, D. Femtosecond laser ablation-ICP-mass spectrometry analysis of a heavy metallic matrix: Determination of

platinum group metals and gold in lead fire-assay buttons as a case study. *J. Anal. At. Spectrom.* **25,** 1259-1267, doi:10.1039/c002746d (2010).
